# Antibodies to citrullinated proteins and differences in clinical progression of rheumatoid arthritis

**DOI:** 10.1186/ar1767

**Published:** 2005-06-14

**Authors:** Annette HM van der Helm-van Mil, Kirsten N Verpoort, Ferdinand C Breedveld, René EM Toes, Tom WJ Huizinga

**Affiliations:** 1 Department of Rheumatology, Leiden University Medical Center, Leiden, The Netherlands

## Abstract

Antibodies to citrullinated proteins (anti-cyclic-citrullinated peptide [anti-CCP] antibodies) are highly specific for rheumatoid arthritis (RA) and precede the onset of disease symptoms, indicating a pathogenetic role for these antibodies in RA. We recently showed that distinct genetic risk factors are associated with either anti-CCP-positive disease or anti-CCP-negative disease. These data are important as they indicate that distinct pathogenic mechanisms are underlying anti-CCP-positive disease or anti-CCP-negative disease. Likewise, these observations raise the question of whether anti-CCP-positive RA and anti-CCP-negative RA are clinically different disease entities. We therefore investigated whether RA patients with anti-CCP antibodies have a different clinical presentation and disease course compared with patients without these autoantibodies. In a cohort of 454 incident patients with RA, 228 patients were anti-CCP-positive and 226 patients were anti-CCP-negative. The early symptoms, tender and swollen joint count, and C-reactive protein level at inclusion, as well as the swollen joint count and radiological destruction during 4 years of follow-up, were compared for the two groups. There were no differences in morning stiffness, type, location and distribution of early symptoms, patients' rated disease activity and C-reactive protein at inclusion between RA patients with and without anti-CCP antibodies. The mean tender and swollen joint count for the different joints at inclusion was similar. At follow-up, patients with anti-CCP antibodies had more swollen joints and more severe radiological destruction. Nevertheless, the distribution of affected joints, for swelling, bone erosions and joint space narrowing, was similar. In conclusion, the phenotype of RA patients with or without anti-CCP antibodies is similar with respect to clinical presentation but differs with respect to disease course.

## Introduction

Autoantibodies directed to citrullinated proteins (e.g. anti-cyclic-citrullinated peptide [anti-CCP] antibodies) are highly specific serological markers for rheumatoid arthritis (RA) that are thought to be directly involved in the disease pathogenesis [1]. Citrullinated proteins are not exclusively located in synovial tissue of RA patients, but can also be found in synovium samples of patients with other inflammatory joint diseases [2] – suggesting that the specificity of anti-CCP antibodies for RA is not due to the expression of citrullinated proteins, but might be the result of an abnormal humoral response. Intriguingly, this antibody response may occur years before any clinical symptoms, as shown by the presence of anti-CCP antibodies several years before the clinical onset of arthritis [3,4]. Furthermore, a proportion of RA patients do not harbour anti-CCP antibodies, suggesting that the presence of anti-CCP antibodies is not obligatory for the development of arthritis or that the pathogenic mechanisms underlying anti-CCP-positive RA and anti-CCP-negative RA are different.

These observations inspired subsequent research addressing the question of whether RA patients with anti-CCP antibodies are different from those who are anti-CCP-negative. We very recently demonstrated in two independent Caucasian populations that the shared epitope encoding HLA-DBR1 alleles is associated with RA in patients with anti-CCP antibodies but not in patients without these antibodies (unpublished data, [5]). These findings are important as they indicate that the shared epitope alleles are not associated with RA as such, but rather with a particular phenotype of the disease.

Given the findings suggesting a pathophysiological role for anti-CCP antibodies in RA and the reported immunogenetic differences between anti-CCP-positive and anti-CCP-negative patients, it is conceivable that anti-CCP-positive RA and anti-CCP-negative RA are different disease entities and thus have different phenotypical properties. Anti-CCP antibodies have been suggested to be associated with more severe radiological outcome [5,6]. To our knowledge, however, a detailed description of the distribution and degree of early symptoms and signs in both patient groups has not been published. Nevertheless, such an analysis is relevant as it might provide novel insight into the putative pathogenic role of anti-CCP antibodies in the aetiology of the disease.

In this study, therefore, we set out to determine whether anti-CCP-positive RA patients and anti-CCP-negative RA patients differ in different aspects of their phenotype: the early symptoms of disease, the findings of physical examination at initial presentation, or the acute phase reactant C-reactive protein at initial presentation. Moreover, we expanded the data on the influence of anti-CCP antibodies on the disease course during 4-year follow-up for the distribution and extent of both inflammation (swollen joints) and radiological joint destruction. We show that the phenotype of RA patients with or without anti-CCP antibodies is similar with respect to clinical presentation but differs with respect to disease course.

## Patients and methods

### Patients

An Early Arthritis Clinic was started in 1993 at the Department of Rheumatology of the Leiden University Medical Center, the only referral centre for rheumatology in a health care region of about 400,000 inhabitants in the western part of The Netherlands [7]. General practitioners were encouraged to refer patients directly when arthritis was suspected. Referred patients could be seen within 2 weeks and were included in the programme when the physician's examination of the patients revealed arthritis and the symptoms had lasted less than 2 years.

At the first visit the rheumatologist answered a questionnaire inquiring about the initial symptoms as reported by the patient (type of initial joint symptoms, localization and distribution of initial joint symptoms, presence of morning stiffness). Patients rated their global assessment of disease activity on a visual analogue scale (0–100). The Health Assessment Questionnaire, a self-assessed questionnaire asking about the ability of the patient to perform several daily activities over the past week, was used to obtain an index of disability. A tender joint count and a swollen joint count [8,9] were performed on entering the study and yearly thereafter. For the tender joint count, each joint was scored on a 0–3 scale with 3 being maximal tenderness (0 = no tenderness, 1 = pain on pressure, 2 = pain and winced, and 3 = winced and withdrew). For the swollen joint count, the individual joints were scored on a 0–1 scale (0 = no swelling, and 1 = swelling).

At inclusion, blood samples were taken from every patient for routine diagnostic laboratory screening including C-reactive protein and were stored to determine antibodies to CCP2 at a later time point. The anti-CCP2 antibody ELISA (Immunoscan RA Mark 2; Euro-diagnostica, Arnhem, The Netherlands) was performed according to the manufacturer's instructions with a cut-off value of 25 units.

More than 1600 early arthritis patients are presently included in the Early Arthritis Clinic cohort and have a follow-up of at least 1 year. A total of 454 patients fulfilled the diagnosis of RA according to criteria of the 1987 American College of Rheumatology 1 year after inclusion in the study. The treatment of the patients in our longitudinal cohort study is characterized by a secular trend. The 122 RA patients (61 anti-CCP-negative and 61 anti-CCP-positive) included between 1993 and 1995 were treated initially with analgesics and subsequently with chloroquine or salazopyrine if they had persistent active disease (delayed treatment). The 135 (70 anti-CCP-negative and 65 anti-CCP-positive) RA patients included between 1996 and 1998 were promptly treated with either chloroquine or salazopyrine (early treatment) (for further description, see [10]). The 197 RA patients (97 anti-CCP-negative and 100 anti-CCP-positive) included after 1998 were promptly treated with either methotrexate or salazopyrine (early treatment).

The rheumatologists that treated the patients were not aware of the anti-CCP status of their patients because anti-CCP antibodies were not routinely determined at inclusion but were assessed for research purposes years after inclusion using stored serum samples. Patients gave their informed consent and the local Ethical Committee approved the protocol.

### Radiographic progression

Radiographs of the hands and feet were made at baseline, at 1 year and yearly thereafter. For 138 patients a complete radiological follow-up was available for 4 years. Inherent to an inception cohort, not all included patients had already completed 4 years of follow-up. Radiographs were scored using the Sharp–van der Heijde method [11]. The rheumatologist that scored the radiographs was blinded to the clinical data and was unaware of the study question. The distribution of radiological destruction of the small joints was studied by comparing the erosion score and joint space narrowing score of the metacarpophalangeal (MCP) and proximal interphalangeal (PIP) joints of the hands.

### Statistical analysis

Differences in means between groups were analysed with the Mann-Whitney test or the *t *test when appropriate. Proportions were compared using the chi-square test. In the analysis of the tender joint count and the swollen joint count, the scores for the left and right joints were summed for each joint location. Furthermore, the scores for the individual MCP joints were summed, as well as the scores for the metatarsophalangeal joints and the interphalangeal joints of the hands and feet. For the 138 RA patients with complete 4-year radiological follow-up, the swollen joint count, the erosion score and the joint space narrowing score were determined for the individual MCP and PIP joints of the hands at inclusion and at 2 and 4 years follow-up, and are expressed as the mean with the 95% confidence interval (CI).

The distribution and degree of radiological destruction and swelling of these joints was studied by comparing the variance of these scores for the individual joints. The 95% CI was used as a measure of variance; as the number of observations in this study is constant (138 patients at all time points during 4 years of follow-up), the extent of the CI reflects the degree of variance. Correlations between joint swelling and erosion score or joint space narrowing score were determined for each MCP and PIP joint of the hands using the Spearman correlation test. The Statistical Package for Social Sciences, version 12.0.1 (SPSS Institute, Chicago, IL, USA) was used to analyse the data. In all tests, *P *< 0.05 was considered significant.

## Results

### Early symptoms of disease

In total 454 patients fulfilled the American College of Rheumatology criteria for RA; 228 of these patients had anti-CCP antibodies and 226 patients had no anti-CCP antibodies at inclusion. Patient characteristics and the type, localization and distribution of initial disease symptoms are presented in Table 1. In both groups, 13% of patients reported no morning stiffness. In the patients that did experience morning stiffness, the mean duration in the anti-CCP-negative patients and anti-CCP-positive patients was similar at 118 min and 123 min, respectively. In both groups symptoms started with pain and swelling, predominantly symmetrical and in the small joints of the hands and feet.

In the statistical analysis without correction for multiple testing, one difference in initial presentation between the two groups was observed: in anti-CCP-positive patients symptoms started more often at both upper and lower extremities than in anti-CCP-negative patients (20% vs 11%, respectively; *P *< 0.05). Given the marginal *P *value, which was not significant after correction for multiple testing, this finding was not considered a relevant difference. The mean patients' rated global disease activity on a visual analogue scale was not significantly different between the two groups. Likewise, the functional ability measured by the Health Assessment Questionnaire score was similar in both groups. In conclusion, there are no fundamental differences in the early symptoms of disease between anti-CCP-positive RA patients and anti-CCP-negative RA patients.

### Findings at physical examination at initial presentation

In each of the 454 patients a tender joint count and a swollen joint count were performed at inclusion. The mean tender joint count per joint is presented in Table 2. There were no significant differences between RA patients with and without anti-CCP antibodies. Table 3 presents the mean scores for joint swelling for both anti-CCP-positive and anti-CCP-negative patients, showing no statistical significant differences between the two groups. Anti-CCP-positive RA patients and anti-CCP-negative RA patients therefore cannot be distinguished at presentation by physical examination.

### Acute phase reactant at initial presentation

The mean C-reactive protein level was 29.5 mg/l (standard deviation [SD], 31.5) in the anti-CCP-negative RA patients and was 35.6 mg/l (SD, 37.8) in the anti-CCP-positive RA patients. The mean C-reactive protein level was not significantly different between the two groups (*P *= 0.08).

### Swollen joints at follow-up

The swollen joint count was assessed yearly in the 138 early arthritis patients with complete radiological follow-up for 4 years. These patients had a mean age at inclusion of 53.7 ± 13.9 years, 67% (93 patients) were women, and 54% (74 patients) were anti-CCP-positive. The total number of swollen joints decreased during follow-up. In the anti-CCP-negative patients at inclusion the mean ± SD number of swollen joint was 10.0 ± 7.2; at 2 years and 4 years follow-up the mean ± SD numbers of swollen joints were, respectively, 4.1 ± 6.7 and 3.1 ± 4.2. The mean ± SD number of swollen joints in the anti-CCP-positive group at inclusion was 8.6 ± 5.5; this decreased to 5.2 ± 7.5 and 5.3 ± 6.8 at 2 years and 4 years follow-up, respectively. At 4 years follow-up the total number of swollen joints was significantly higher in the RA patients with anti-CCP antibodies (*P *= 0.01).

**Figure 1:**
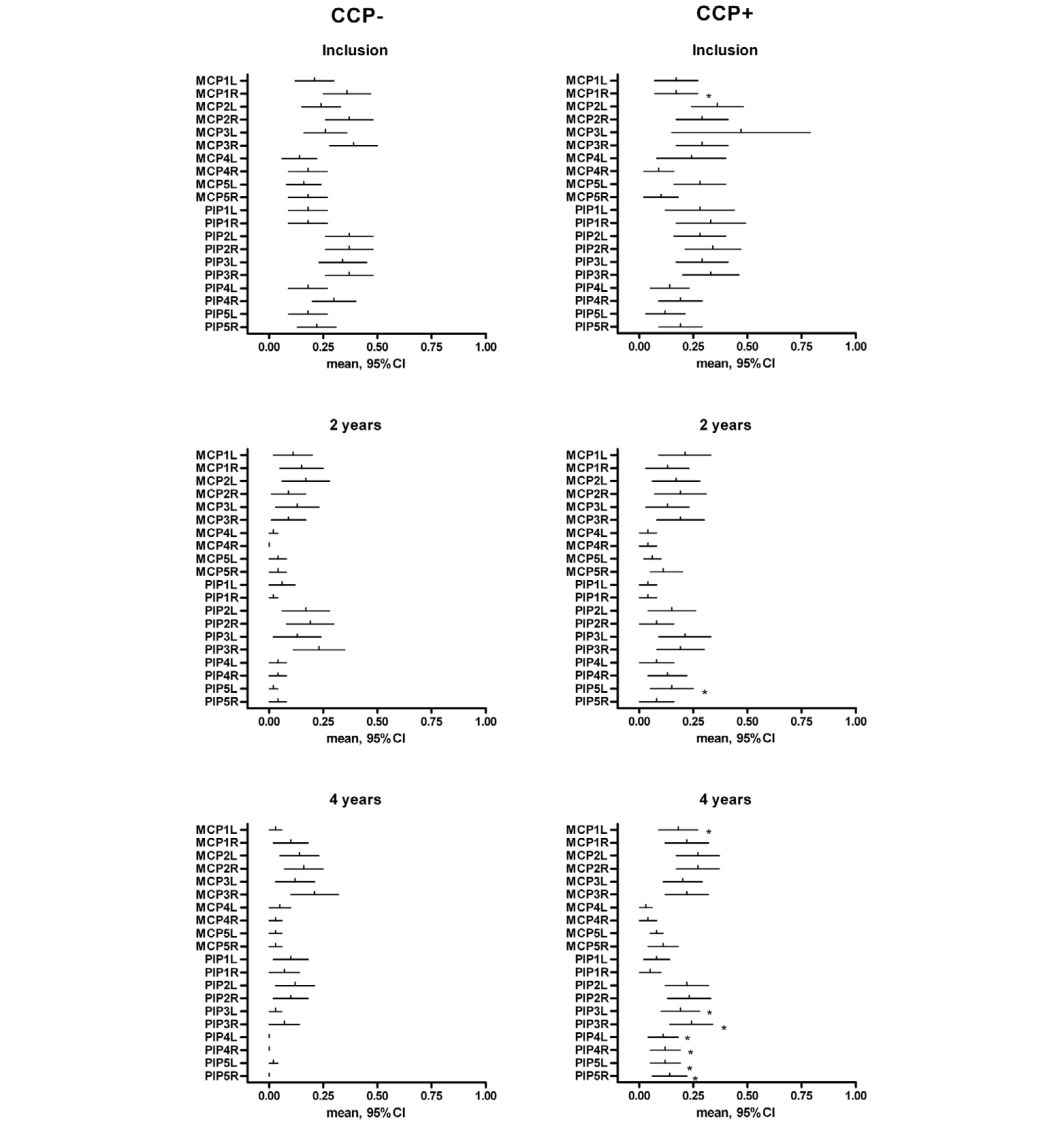
Swelling of the MCP and PIP joints at inclusion and follow-up. Joint swelling (mean and 95% confidence interval [CI]) of the metacarpophalangeal (MCP) and proximal interphalangeal (PIP) joints of the hands at inclusion and at 2 and 4 years follow-up in rheumatoid arthritis patients with (CCP+) and without (CCP-) anti-cyclic-citrullinated peptide antibodies. L, left; R, right.

In addition, the scores for the individual MCP and PIP joints of the hands were compared. Overall the pattern of inflammation of the individual small joints is similar in anti-CCP-negative RA and in anti-CCP-positive RA, as is depicted by the mean and 95% CI of the swollen joint count in Fig. 1. Several individual joints had significantly higher scores in the anti-CCP-positive patients compared with the anti-CCP-negative patients; at inclusion this concerned the first MCP joint on the right side, at 2 years follow-up this concerned the fifth PIP joint on the left side, and at 4 years follow-up this concerned the first MCP, third PIP, fourth PIP and fifth PIP joints on the left side and the third PIP, fourth PIP and fifth PIP joints on the right side (*P *< 0.05). Furthermore, Fig. 1 shows that in both anti-CCP-positive RA patients and anti-CCP-negative RA patients, the second and third MCP joints were more frequently swollen than the other MCP joints. Likewise, in both groups the second and third PIP joints were more frequently affected than the other PIP joints. In conclusion, the pattern of inflammation of the individual small joints of the hand seems similar in anti-CCP-positive and anti-CCP-negative patients; however, particularly at 4 years follow-up some MCP and PIP joints are significantly less frequently swollen in anti-CCP-negative RA patients.

### Radiographic progression

**Figure 2:**
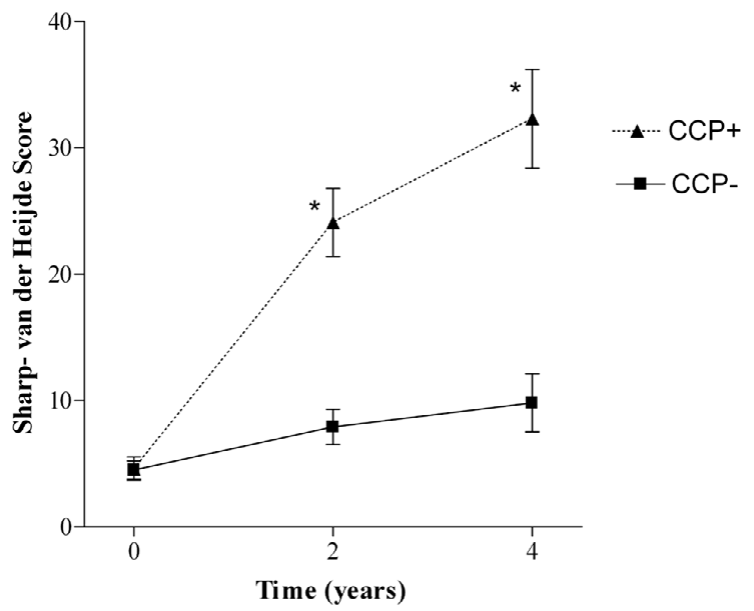
Radiological destruction in patients with and without anti-cyclic-citrullinated peptide antibodies.Total Sharp–van der Heijde scores (mean ± standard error of the mean) at inclusion and at 2 and 4 years follow-up in rheumatoid arthritis patients with (CCP+) and without (CCP-) anti-cyclic-citrullinated peptide antibodies.

In the 138 RA patients with a complete 4-year radiological follow-up, the total Sharp–van der Heijde scores between the RA patients with and without anti-CCP antibodies were compared (Fig. 2). At 2 years and 4 years follow-up, anti-CCP-positive patients had significantly higher radiological scores than anti-CCP-negative patients (*P *< 0.001).

**Figure 3:**
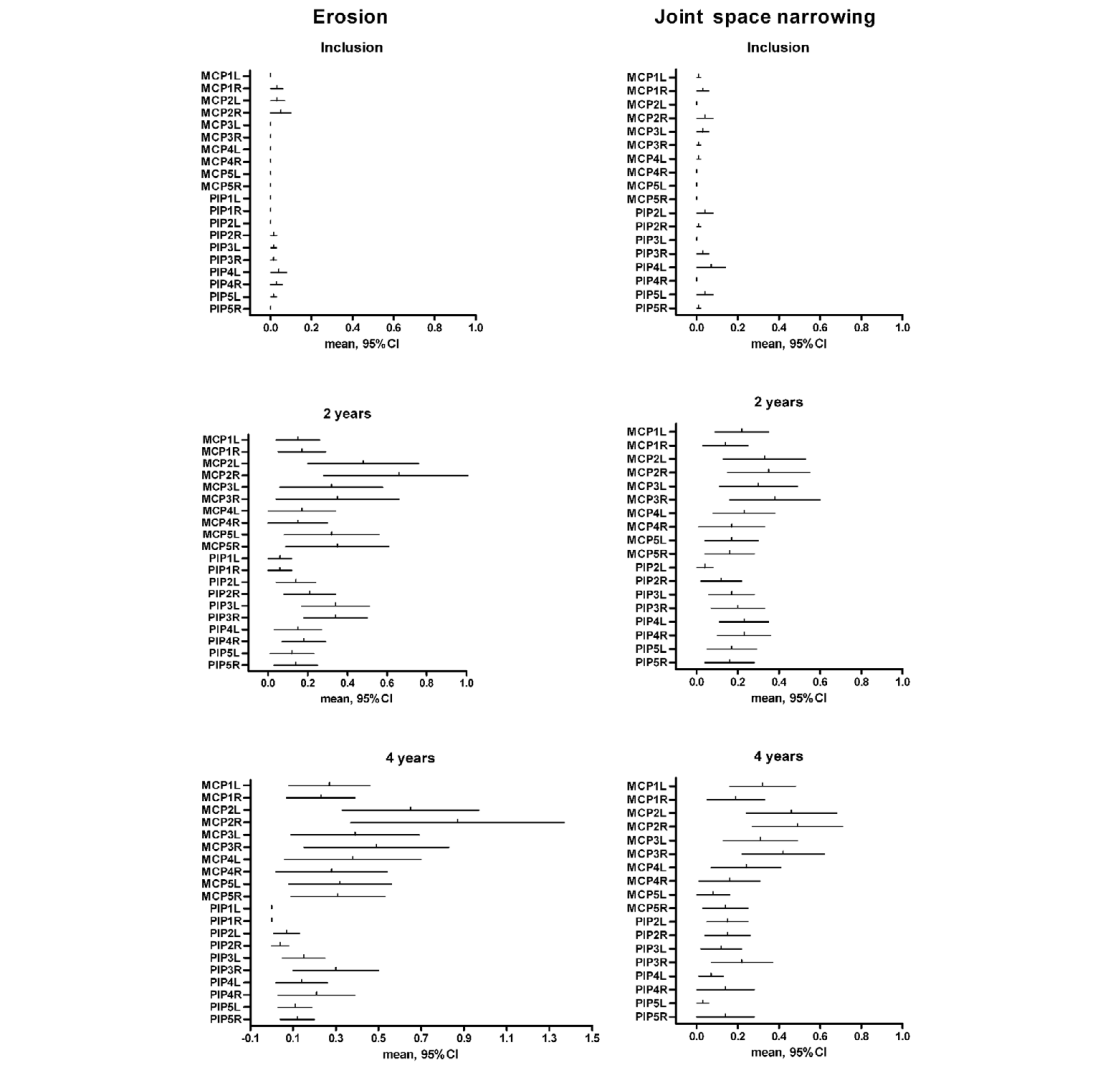
Erosion and joint space narrowing scores of MCP and PIP joints at Inclusion and follow-up. Erosion and joint space narrowing scores of the metacarpophalangeal (MCP) and proximal interphalangeal (PIP) joints of the hands (means and 95% confidence interval [CI]) at inclusion and at 2 and 4 years follow-up in rheumatoid arthritis patients with anti-cyclic-citrullinated peptide antibodies. L, left; R, right.

The distribution of the radiological destruction in the MCP and PIP joints of the hands was further investigated. The erosion scores and joint space narrowing scores of the MCP and PIP joints are depicted in Fig. 3. As the most pronounced radiological destruction was present in anti-CCP-positive patients, the erosion scores and joint space narrowing scores are shown for the RA patients with anti-CCP antibodies. Figure 3 shows that at all time points, of all the MCP joints, the second MCP joints had the highest erosion score, followed by the third MCP joints. Concerning the PIP joints, the highest erosion scores were present in the third and fourth PIP joints. Figure 3 further reveals that the second and third MCP joints are the MCP joints with the highest joint space narrowing scores at all time points during follow-up. The joint space narrowing scores of the PIP joints differ less, but there are slightly higher scores for the third and fourth PIP joints.

The erosion scores and joint space narrowing scores for the patients without anti-CCP antibodies revealed the same distribution as for the anti-CCP-positive RA patients (data not shown). In the anti-CCP-negative patients the values for the mean and 95% CI were lower than in the anti-CCP-positive patients, which is in concordance with the finding of lower total Sharp–van der Heijde scores in anti-CCP-negative RA patients. Correlations between joint swelling and the erosion score and between joint swelling and the joint space narrowing score were determined for each MCP and PIP joint at 4 years follow-up. For all PIP joints and for all MCP joints, except the fourth MCP joints, the erosion score was significantly correlated with joint swelling (*P *< 0.05). The joint space narrowing scores were significantly correlated with joint swelling in all MCP joints except the fourth MCP joint (*P *< 0.05). This implies that at that time point the joints that were the most swollen were also the joints with the most severe radiological destruction.

## Discussion

This study shows that the phenotype of RA patients with or without anti-CCP antibodies does not differ at clinical presentation. In a large, prospective, early arthritis cohort we observed neither a significant difference in the reported first symptoms nor in the signs found in the physical examination at initial presentation between anti-CCP-positive patients and anti-CCP-negative patients. During follow-up, however, anti-CCP-positive RA patients have more swollen joints and show more radiological destruction than anti-CCP-negative RA patients. It is remarkable that at follow-up, in spite of the difference in magnitude of the disease characteristics, the distribution of swollen joints and the distribution of radiological joint space narrowing and bone erosions remains similar for RA patients with and without anti-CCP antibodies. This implies that although different associations with known risk factors are reported for anti-CCP-positive and anti-CCP-negative RA patients, the presence or absence of anti-CCP antibodies is not associated with a distinguishable clinical phenotype at presentation of disease.

Pathophysiologically, this may have implications. It was recently observed that the prominent genetic risk factor HLA class II alleles only associate with susceptibility to RA in the presence of anti-CCP antibodies but not with RA in the absence of these antibodies (unpublished data, [5]). It has been shown in mice that citrullination of arginine in a peptide can lead to a higher binding affinity of that peptide for HLA-DRB*0401, an important shared epitope allele [12], allowing peptide-specific T-cell induction. It can be speculated that also in humans citrullination may improve antigen presentation to CD4-positive T cells and that the genetic background (presence of shared epitope alleles) provides the basis for a citrulline-specific immune reaction.

It has been demonstrated that anti-CCP antibodies occur years before disease onset [3,4]. This latter observation suggests that the induction of disease in anti-CCP-positive RA patients occurs years before presentation. The current study, however, shows that the age of onset of clinical disease is similar in RA patients with and without anti-CCP antibodies.

The risk factors such as HLA alleles differ between anti-CCP-negative RA and anti-CCP-positive RA [5]. Although differences in risk factors presume different pathophysiological pathways for anti-CCP-positive RA and anti-CCP-negative RA, the initial phenotypical presentation of both patient groups is similar and is characterized by a symmetric polyarthritis of the same small joints. At follow-up the clinical phenotype remains comparable with regard to joint distribution, but the anti-CCP-positive patients have more inflamed joints and once there is inflammation also have more rapid joint destruction. This leads to a pathophysiological model in which one or more triggers lead to arthritis in similar joints in anti-CCP-positive patients and anti-CCP-negative patients. Antigens are subsequently citrullinated during inflammation; in the presence of anti-CCP antibodies the inflammation is aggravated, resulting in more severe radiological destruction. Further studies are needed to add insight into the pathogenic role of circulating anti-CCP antibodies in anti-CCP-positive RA and to unravel the risk factors associated with anti-CCP-negative RA.

In a study by Kastbom and colleagues [13] several baseline disease characteristics of anti-CCP-positive RA patients and anti-CCP-negative RA patients were compared. This study observed no significant differences in baseline total swollen joint count, in C-reactive protein levels or in the Disease Activity Score (DAS)28 score between RA patients with and without anti-CCP antibodies, but showed a positive correlation between the number of fulfilled American College of Rheumatology criteria and the frequency of anti-CCP positivity [13]. Furthermore, in that study anti-CCP-positive individuals were more often treated with disease-modifying antirheumatic drugs than were anti-CCP-negative patients [13].

Although in the present study secular trends in the initial treatment strategies with disease-modifying antirheumatic drugs were present, these trends yielded the same effect for the anti-CCP-positive and anti-CCP-negative RA patients. Furthermore, the rheumatologists that treated the patients were not aware of the anti-CCP status of their patients. The more severe disease course in patients with anti-CCP antibodies is therefore probably not due either to a more delayed treatment of these patients or to confounding by treatment adapted to the anti-CCP status. We cannot exclude the fact that during follow-up the anti-CCP-positive patients that had more inflamed joints received more aggressive treatment. In the case of a more aggressive treatment during follow-up in anti-CCP-positive patients, however, this did not prevent the development of more severe radiological destruction in the RA patients with anti-CCP antibodies. The finding that the swollen joint count decreased during follow-up is probably due to the fact that patients were not treated with disease-modifying antirheumatic drugs at inclusion.

The sensitivity of anti-CCP2 antibodies for RA is reported to vary between 39% and 80% [14,15]. The present study measured anti-CCP2 levels at inclusion (a very early stage of the disease) and reports a relatively low percentage (50%) of RA patients with anti-CCP antibodies. As cyclic-citrullinated peptide measurements were not repeated during follow-up, we cannot exclude that some RA patients that were anti-CCP-negative at inclusion have become anti-CCP-positive at a later stage in the disease. A relatively low prevalence of anti-CCP antibodies in early arthritis patients has been described previously [14].

The present study shows that the second and third MCP joints have the highest erosion scores as well as the highest joint space narrowing scores and are, of all the MCP joints, the most frequently swollen. Although the present study was not designed to study the correlation between inflammation and destruction, the observed similarity in joints that are affected by swelling, erosions and joint space narrowing supports the concept that, in general, the mechanisms leading to clinical inflammation and radiological destruction are related.

The present study includes a detailed description on the distribution of affected joints in RA and shows that the MCP joints of the second and the third digits are most frequently inflamed and destroyed. Although to our experience rheumatologists generally feel that the joints of the second and third digits are more frequently inflamed than other joints of the hands, to our knowledge this phenotypic characterization has not been frequently described.

## Conclusion

The present study shows that, although separate risk factors for anti-CCP-positive RA and anti-CCP-negative RA have been recently described, the clinical presentation of RA patients with or without anti-CCP antibodies is not different. Patients with anti-CCP antibodies develop a more severe disease course with more radiological destruction compared with RA patients without these autoantibodies. Nonetheless, the distribution of affected joints is also similar at follow-up.

## Abbreviations

anti-CCP = anti-cyclic-citrullinated peptide antibodies; CI = confidence interval; MCP = metacarpophalangeal; PIP = proximal interphaleangeal; RA = rheumatoid arthritis; SD = standard deviation.

## Competing interests

The author(s) declare that they have no competing interests.

## Authors' contributions

AHMvdHvM collected clinical data, carried out the statistical analysis and drafted the manuscript. KNV performed the anti-CCP2 ELISA and helped to draft the manuscript. FCB participated in the performance of and the coordination of the study. REMT participated in the design of the study and reviewed the draft manuscript. TWJH participated in the design of the protocol, contributed to the coordination of the study, supervised the statistical analysis and reviewed the draft manuscript. All authors read and approved the final manuscript.

## Figures and Tables

**Table 1:** Characteristics of the early symptoms in rheumatoid arthritis patients with and without anti-cyclic-citrullinated peptide (anti-CCP) antibodies

	Anti-CCP-negative (*n *= 228)	Anti-CCP-positive (*n *= 226)
Female [*n *(%)]	147 (64%)	150 (66%)
Age at inclusion (mean ± standard deviation)	57 ± 17	55 ± 16
Morning stiffness		
No [*n *(%)]	30 (13%)	30 (13%)
Yes (min) (mean ± standard deviation)	118 ± 138	123 ± 128
Type of initial joint symptoms [*n *(%)]^a^		
Pain	208 (91%)	205 (91%)
Swelling	146 (64%)	135 (60%)
Stiffness	106 (46%)	85 (38%)
Function loss	64 (28%)	57 (25%)
Redness or increased surface temperature of joints	19 (8%)	26 (12%)
Localization of initial joint symptoms [*n *(%)]		
Small joints of hands and/or feet	105 (46%)	112 (50%)
Large joints	54 (24%)	50 (22%)
Both small and large joints	63 (28%)	59 (26%)
Unknown	6 (2%)	5 (2%)
Localization of initial joint symptoms [*n *(%)]		
Upper limbs	114 (50%)	86 (38%)*
Lower limbs	72 (32%)	77 (34%)
Both upper and lower limbs	25 (11%)	45 (20%)*
Unknown	18 (8%)	18 (8%)
Localization of initial joint symptoms [*n *(%)]		
Symmetric	145 (64%)	130 (58%)
Asymmetric	71 (31%)	83 (37%)
Unknown	10 (4%)	13 (6%)
VAS patients' rated global disease activity (0–100)	51.3 ± 39.9	46.7 ± 28.2
HAQ score (mean ± standard deviation)	1.0 ± 0.7	1.0 ± 0.7

VAS, visual analogue scale. HAQ, Health Assessment Questionnaire.

^a^Patients can have both swelling and pain at the start of the symptoms and therefore the total can add to more than 100%.

* *P *< 0.05, anti-CCP-positive versus anti-CCP-negative.

**Table 2:** Tender joint count at inclusion in rheumatoid arthritis patients with and without anti-cyclic-citrullinated peptide (anti-CCP) antibodies

	Anti-CCP-negative (*n *= 228)	Anti-CCP-positive (*n *= 226)
Temporomandibular joints	0.01 ± 0.41	0.08 ± 0.36
Sternoclavicular joints	0.23 ± 0.76	0.12 ± 0.47
Acromioclavicular joints	0.31 ± 0.63	0.55 ± 0.79
Shoulder joints	0.85 ± 1.5	0.86 ± 1.4
Elbow joints	0.42 ± 0.99	0.35 ± 0.81
Wrist joints	0.94 ± 0.94	0.80 ± 0.93
Metacarpophalangeal joints	4.3 ± 4.3	3.5 ± 3.4
Proximal interphalangeal joints of the hands	3.2 ± 3.6	3.3 ± 3.4
Distal interphalangeal joints of the hands	1.3 ± 2.4	1.2 ± 2.2
Hip joints	0.18 ± 0.73	0.11 ± 0.54
Knee joints	0.54 ± 0.88	0.59 ± 0.90
Ankle joints	0.41 ± 0.92	0.53 ± 1.1
Subtalar joints	0.31 ± 0.72	0.52 ± 0.76
Midtarsal joints	0.21 ± 0.40	0.18 ± 0.58
Metatarsophalangeal joints	4.2 ± 3.4	4.1 ± 3.7
Interphalangeal joints of the feet	0.91 ± 1.8	1.4 ± 3.2
Total Ritchie articular index score	10.4 ± 8.2	10.2 ± 8.0

Tenderness was scored per joint on a 0–3 scale: 0 = no tenderness, 1 = pain at pressure, 2 = pain and winced, and 3 = winced and withdrew. The scores for the metacarpophalangeal joints were summed, as were the scores for the metatarsophalangeal joints and the interphalangeal joints of the hands and feet. The scores for the left joints and the right joints were summed. The summed scores were divided by the total numbers of patients; the resulting mean ± standard deviation is presented. There were no statistical differences between patients with and without anti-CCP antibodies.

**Table 3:** Joint swelling at inclusion in rheumatoid arthritis patients with and without anti-cyclic-citrullinated peptide (anti-CCP) antibodies

	Anti-CCP-negative (*n *= 228)	Anti-CCP-positive (*n *= 226)
Temporomandibular joints	0.01 ± 0.10	0.02 ± 0.18
Sternoclavicular joints	0.08 ± 0.34	0.04 ± 0.22
Acromioclavicular joints	0.06 ± 0.24	0.03 ± 0.17
Shoulder joints	0.08 ± 0.30	0.12 ± 0.40
Elbow joints	0.22 ± 0.54	0.20 ± 0.49
Wrist joints	1.0 ± 0.89	1.0 ± 0.90
Metacarpophalangeal joints	3.2 ± 3.0	2.2 ± 2.2
Proximal interphalangeal joins of the hands	2.6 ± 3.1	2.0 ± 1.8
Distal interphalangeal joints of the hands	0.32 ± 0.60	0.21 ± 0.60
Knee joints	0.46 ± 0.74	0.49 ± 0.74
Ankle joints	0.31 ± 0.67	0.34 ± 0.63
Subtalar joints	0.24 ± 0.61	0.21 ± 0.55
Metatarsophalangeal joints	1.6 ± 2.2	1.8 ± 2.4
Interphalangeal joints of the feet	0.06 ± 0.24	0.18 ± 0.58
Total number of swollen joints	10.0 ± 7.2	8.6 ± 5.5

Swelling was scored for each joint on a 0–1 scale: 0 = no swelling, and 1 = swelling. The scores for the metacarpophalangeal joints were summed, as were the scores for the metatarsophalangeal joints and the interphalangeal joins of the hands and feet. The scores for the left joints and the right joints were summed. The summed scores were divided by the total numbers of patients; the resulting mean ± standard deviation is presented. There were no statistical differences between patients with and without anti-CCP antibodies.
